# Congenital Toxoplasmosis: A Plea for a Neglected Disease

**DOI:** 10.3390/pathogens7010025

**Published:** 2018-02-23

**Authors:** Martine Wallon, François Peyron

**Affiliations:** 1 Hospices Civils de Lyon, Institut de Parasitologie et Mycologie Médicale, Hôpital de la Croix Rousse, F-69317 Lyon, France; martine.wallon@chu-lyon.fr; 2 Waking team, Lyon Neuroscience Research Center, INSERM U1028-CNRS UMR 5292, F-69008 Lyon, France

**Keywords:** congenital toxoplasmosis, mass screening, antenatal treatment efficacy, cost effectiveness

## Abstract

Maternal infection by *Toxoplasma gondii* during pregnancy may have serious consequences for the fetus, ranging from miscarriage, central nervous system involvement, retinochoroiditis, or subclinical infection at birth with a risk of late onset of ocular diseases. As infection in pregnant women is usually symptomless, the diagnosis relies only on serological tests. Some countries like France and Austria have organized a regular serological testing of pregnant women, some others have no prenatal program of surveillance. Reasons for these discrepant attitudes are many and debatable. Among them are the efficacy of antenatal treatment and cost-effectiveness of such a program. A significant body of data demonstrated that rapid onset of treatment after maternal infection reduces the risk and severity of fetal infection. Recent cost-effectiveness studies support regular screening. This lack of consensus put both pregnant women and care providers in a difficult situation. Another reason why congenital toxoplasmosis is disregarded in some countries is the lack of precise information about its impact on the population. Precise estimations on the burden of the disease can be achieved by systematic screening that will avoid bias or underreporting of cases and provide a clear view of its outcome.

## 1. Introduction

The ubiquitous parasite *Toxoplasma gondii* (*T. gondii*) has been known for 80 years for its potential to cause severe damage when acquired *in utero* as a consequence of a maternal infection during pregnancy [1]. A large body of research has been published on the fundamental aspects of the parasite and on how it interacts with its large range of possible hosts. Reports regarding human toxoplasmosis and their consequences are also numerous. Recent publications revealed that serological screening followed by treatment during pregnancy significantly reduces the risk and severity of fetal infection (see below). However, there is no international consensus for the surveillance of the disease in pregnant women. Attitudes and habits still vary widely regarding the management of maternal and congenital infections. In France, an antenatal program is mandatory, in the United States of America (USA) it is not. Between these two opposite attitudes recommendations widely vary according to countries, and prenatal surveillance by means of regular serological tests is still controversial. This situation is becoming difficult for pregnant women and care providers both looking for precise information and guidelines. In this paper, we present the pros and cons regarding regular testing of pregnant women and try to convince the reader to drive congenital toxoplasmosis (CT) out of this shadow situation.

## 2. Congenital Toxoplasmosis

*T. gondii* infection during pregnancy can have devastating consequences in the fetus ranging from stillbirth, hydrocephalus, and ocular damage. Some of the infected newborns present with a sub clinical infection, but are at risk of developing ocular lesion at any time. Such retinal lesion can reactivate and their impact on sight depends on their localization with respect to the macula. Severity of fetal infection depends on different factors.

Parasite genotype seems to play a role in virulence in human infections. In Europe, especially in France, parasitic population is clonal with a largely predominant type II of moderate virulence in immunocompetent patients [2]. In Brazil, type II is rare and clonal lineages named BR I, BR II, BR III, and BR IV have been isolated in animals [3]. Atypical genotypes have also been reported in humans [4]. Ocular lesions in CT appear more severe in Brazil than in Europe [5]. Atypical strains from French Guyana lead to severe infections in immunocompetent patients [6]. In the USA, type II strains were associated with a different type of hydrocephalus when compared to non-type II strains [7]. A second factor influencing risk and severity of foetal infection is the age of pregnancy at maternal infection. Data from cohorts of pregnant women routinely screened during pregnancy showed that for early maternal infection, foetal infection is rare but severe, and conversely for late maternal infection, risk of foetal infection is high, but generally subclinical [8,9,10]. Interestingly, infection before pregnancy, marked by a positive serology, induces an efficient immunoprotection for the forthcoming pregnancy.

Only 30% of maternal infections induce clinical manifestations, suggestive of acute toxoplasmosis. Signs are nonspecific: fatigue, lymph nodes, especially in the cervical region, and a bout of fever [11]. Diagnosis relies essentially on serological tests. The best marker of acute infection is a true seroconversion, with IgG changing from negative to positive. More frequently, positive IgG and IgM antibodies are already detected at the first test. As IgM can last for years, their presence is not a reliable marker of recent infection. High IgG avidity and stable level of IgG over a four-week interval can rule out an acute infection. It should be kept in mind that IgG avidity maturation can be delayed. Therefore the diagnosis of an acute infection cannot reliably rely on a low IgG avidity index and presence of IgM. When the first sample is drawn late in pregnancy ruling out a per-gestational infection is difficult. In many cases, samples should be referred to a reference laboratory for proper interpretation.

Regular testing of susceptible women (i.e. seronegative) can lead to rapidly recognizing an acute infection and eventually performing antenatal diagnosis and promptly offering an adapted treatment in order to either prevent the foetal infection or abate its sequelae [9]. Additionally, knowing the time when maternal infection has occurred allows for accurately estimating the risk and the severity of foetal infection and for offering better counselling to parents-to-be [8].

Whether or not systematic screening of pregnant women should be implemented is certainly the most disputed issue in the field of CT. Mass screening programs have been implemented for more than 25 years at no costs for the patients in France and in Austria, with monthly or two monthly retests, respectively. They are also organized in other European countries, including Italy [12] and Slovenia [13]. Many countries, like the US, have no screening program and pregnant women are rarely tested for toxoplasmosis unless presenting with clinical signs, being at risk of infection or presenting abnormal foetal ultrasound. This strategy misses half of the mothers who give birth to congenitally infected babies [14].

Why do many countries disregard CT?

## 3. Should We Screen Pregnant Women for Toxoplasmosis?

Mass screening should fulfill some prerequisites proposed by World Health Organization [15]. Among them the most stringent are [16]:

- the disease should represent a major cause of death or have a substantial prevalence in the population;
- treatment should be available for latent or early stage that improve the outcome;
- the screening tests should have a good scoring;
- screening should be cost effective and acceptable to the population;
- facilities for diagnosing and treatment should be available;
- there should be agreement among clinical guidelines on whom to treat.

Opponents argue that mass screening does not fulfill at least three of these criteria and that the incidence and the impact of CT in the population do not justify a systematic screening.

### 3.1. The Cons

Indeed, since the past decades the seroprevalence of toxoplasmosis has decreased in many countries. In France, in the general population, it has dropped from 80% in 1960 to 36.7% in 2010 [17]. In the USA, the same decline has been reported with a seroprevalence among women of child bearing age decreasing from 14.9% in a 1988–1994 NHANES survey to 9.1% in a 2009–2010 NHANES survey [18,19]. Data is lacking for other countries, but seroprevalence displays a large geographic variation. The lowest rates are found in Asia and North America, the highest in South America [20].

This trend poses a real problem for countries when considering a systematic surveillance of CT in pregnancy as the number of susceptible women (i.e. non immune) is regularly increasing and the number of congenitally infected newborns is dropping. Nevertheless, in countries with no surveillance program, it is hardly possible to have an accurate evaluation of the impact of CT in the population in term of burden of the disease, patients placed in institutions, quality of life, undue pregnancy terminations due to anxiety for pregnant women abruptly told of a positive serology for a disease that they have never been informed of is positive, and their fetus is at risk of presenting with neurological lesions. Moreover it has to be born in mind that some rarer metabolic diseases, like phenylketonuria, are screened in most states of the USA [21].

This situation raises the cost-benefit aspects of mass screening, which will be addressed below. The lack of randomized controlled study, demonstrating the efficacy of antenatal treatment for preventing or abating sequelae of fetal infection is the most important point raised by opponents to mass screening. From an academic point of view, we have no direct evidence supporting antenatal treatment efficacy. However, given the numerous observational studies reporting favorable outcomes (see below), a prospective randomized controlled study appears unethical [21].

Additionally, as stated by Baicker and Chandra [22] ”there is a key difference between no evidence of effect and evidence of no effect”.

### 3.2. The Pros

For mass screening advocates, there is lot of evidences supporting prenatal program.

As said before, numerous reports in literature suggest that treatment of toxoplasmosis during pregnancy reduces the risk of fetal infection and sequelae [9,10,23,24,25,26]. A meta-analysis of individual patient data on congenital toxoplasmosis (SYROCOT) demonstrated that transmission rate was significantly reduced by half when treatment was introduced three weeks *versus* eight weeks after the estimated date of maternal infection [26]. Data from the French Lyon cohort study reported that when monthly screening was implemented in 1992 the risk of foetal infection fell significantly when compared to screening before 1992 (for example at 26 weeks of gestation the risk dropped from 59.6% to 46.6%; *p* = 0.038) [9]. The same study reported that when PCR on amniotic fluid was routinely introduced in 1995 (i.e. infected foetuses were treated earlier with pyrimethamine and sulfadiazine) the risk of developing clinical signs in children followed for three years dropped significantly (OR: 0.59, 95%CI: 0.4 to 0.89, *p* = 0.012) and the odds of severe neurologic sequelae or death in infants with CT was also significantly lower (OR: 0.24; 95% CI: 0.07–0.71) [9]. Kieffer et al. found that a shorter delay between maternal infection and treatment onset significantly reduced the risk of ocular lesion during a three-year follow up [24]. A European based study also demonstrated that antenatal screening and treatment significantly reduced the occurrence of severe neurologic sequelae or death [10]. Gras et al. reported a significant reduction of cerebral calcifications when treatment was given within four weeks after maternal infection [27]. Prusa et al. recently reported that antenatal treatment reduces the risk of mother to child transmission when compared with those without treatment [25]. Differences in outcomes were also significant between two cohorts of CT, in the USA and France that were caused by the same type of strain (Type II), with rates of hydrocephalus of 31% and 0.8%, respectively [28].

Guidelines for monitoring maternal infections and congenitally infected infants are available [29], with minor differences mainly due to lack of availability of some drugs. Safety of pyrimethamine-based treatments is also a concern. However, in a systematic review Ben-Harari et al. did not report Steven-Johnson syndrome in either 737 pregnant women or 929 infants/children treated for toxoplasmosis [30].

As pointed out by Maldonado et al. such surveillance program depends on the incidence of the disease and the burden of CT in a given population [31]. In 2015, WHO ranked *T. gondii* the third most important cause of foodborne disease [32]. Using DALY (disability-adjusted life years) as metric, burden of CT was evaluated at 123 DALYs in Denmark [33], while it was estimated at 2.251 DALYs in the Netherlands [34]. Although this difference can be due to a lower incidence of CT in Denmark it can also be explained by difference in Disability Weight attributed to different impairments like chorioretinitis in the two studies. This discrepancy highlights the lack of precise and comparable information available with respect to the impact of CT. Despite its declining incidence, CT should be considered as a concern with respect to a public health point of view. In the United States, toxoplasmosis was found to be the second leading cause of death and the fourth leading cause of hospitalization attributable to foodborne diseases [30].

Early evaluation concluded that mass screening was not cost-effective, but in their analysis, the authors assumed that screening tests have a low specificity [35]. Given the good performances of recent serological tests (see below), this assumption should be reconsidered. A decision-analytic economic model published in 2011 stated that mass screening is cost-effective in the USA providing a cost of $12 per test [36]. In Austria, where prenatal screening is mandatory, a decision-analytic model comparing lifetime societal costs with and without screening concluded that the program of prenatal screening was cost-saving [37]. Nevertheless, controlling the price of tests should be a priority. Accurate, cheap, and easy to use rapid diagnostic tests are available [38,39]. Such tests will certainly help to promote the screening of pregnant women even in low and middle income countries. Given the body of data about antenatal treatment efficacy, another less academic manner to address this issue is to ask policy makers the amount of money they agree to invest in mass screening for treating an infected fetus. Another reason for supporting mass screening is the availability of accurate tests for detecting maternal seroconversion and fetal infection. In the context of a serological retesting program, knowledge of a previous negative test helps in recognizing the conversion in IgG as a clear sign of acute infection. A rise in IgG between two consecutive samples in the presence of high titers of IgM would otherwise have to be observed to confirm a per-partum *Toxoplasma* infection and make a rapid onset of treatment after infection possible. The presence of IgM associated with IgG on a single serum is indeed an unreliable predictor of an acute infection because of the possibility of IgM to persist for several months or years after the infection. The same holds for the avidity index that might remain low for several months or a year [40]. In this setting, precise information on the age of pregnancy at maternal infection provides reliable estimation of the risk and severity of fetal infection, influencing the decision to perform amniocentesis and to treat the newborn at birth [8]. In a context with no surveillance program, the required serological confirmation of an acute infection is less straightforward. When the first test is performed late in pregnancy, either at women’s request or because of clinical signs evoking a *T. gondii* infection, the expertise of a reference laboratory is required if IgM are detected, alone or with IgG.

Antenatal diagnosis has tremendously progressed. The analysis of amniotic fluid by PCR offers an excellent decision tool regarding treatment, as confirmed by a recent meta-analysis [41]. Amniotic fluid should be sampled after 18 weeks of gestation and at least four weeks after the date of maternal infection. Polymerase chain reaction assays for the detection of *T. gondii* DNA are close to 100% specific [42]. When PCR is positive, spiramycine, initially given as soon as maternal infection is diagnosed, is discontinued and is switched to a combination of sulfadiazine and pyrimethamine. This combination of drug is considered as the most effective against *T. gondii*.

Ultrasound surveillance should be carried out every two to four weeks, according to the gestational age at maternal infection and to PCR results on amniotic fluid. The most characteristic ultrasound signs concern the brain: calcifications are the most common and appear as several millimeters in diameters in the parenchyma, and in a periventricular area and basal ganglia. They are due to focal brain necrosis that is associated with calcifications. Their appearance differs from those that are observed in Zika or CMV [43]. They may be associated with ventricular dilatation, which is generally symmetrical and rapidly progression, and of poor long term prognosis. Less specific signs, such as hepatomegaly, ascitis, pericardial effusion, hyper echogenic fetal bowel, and increase in the size of the placenta can also be observed. Antenatal MRI may be indicated, especially in lesions occurring after a first trimester infection.

There are other advantages of systematic screening:

One of them is the possibility to rapidly inform pregnant women about their immunological status with regard to toxoplasmosis and to advise on how to avoid *Toxoplasma* infection those who are found to be non-immune. As advocated by Pereboom et al., information should focus on the overall avoidance of risky behaviors and on promoting healthy lifestyle, rather than on details on the parasite and its cycle [44]. Surveys performed among internists, obstetricians, midwives, and nurses have revealed the need for health professionals to be better trained. Their knowledge should be regularly checked and updated to help them answer questions, reinforce correct behaviors, and identify mistakes or unnecessary efforts. They found that key advice was often overlooked, such as washing hands and avoiding gardening without gloves. Moreover, the promotion of good hand hygiene and of avoiding undercooked meat can also help to prevent other pathogens. (See [29] for an overview of preventive measures).

Screening for maternal infections also allows investigating newborns at birth and submitting them to a complete work up [29]. There are no guidelines for treatment of CT at birth. Current regimens are based on the combination of antifolate and sulfonamides. Generally treatment is given for a year (see [29] for different regimens). Another advantage is the systematic follow-up of all CT, and not only those with a patent infection, which avoids a biased view on the long-term evolution of the disease. One of the most distressing questions that parents ask doctors is about the long term outcome of a congenitally infected newborn. When gross neurological malformations are observed at birth, the outcome is rather poor and some of these infants have to be placed in an institution. Post-natal treatment appears to improve the outcome, even in the absence of antenatal treatment [45]. In settings where infants receive ante- and post-natal treatment, CT is rarely severe, but patients are at risk of new or relapsing ocular lesions during their life. In a cohort of 485 cases of CT, first lesions appeared at a median age of 3.1(0.0–20.7) years. In 33.8% of cases, recurrence or first ocular lesion appeared up to 12 years [46]. Visual performance using the VF14 questionnaire was evaluated on 126 adults presenting with CT and monitored since birth. Among them, 58.8% presented with at least one ocular lesion with a foveal localization in 15.7% of cases. Visual function was slightly impaired with a VF14 global score of 97.3 (out of 100) [47]. During the last decade, numerous publications have dealt with possible link between patients with positive toxoplasmic serology and numerous neuropsychological disorders such as schizophrenia, bipolar disorders, obsessive compulsive disorders, epilepsy, recurrent migraines, Parkinson’s, aggressive behaviors and traffic accidents [48]. However, as pointed out by Del Grande et al. [49] this topic remains controversial without cause-effect studies. A questionnaire study on 126 congenitally infected adults was conducted for evaluating quality of life with a Psychological Wellbeing Index questionnaire, addressing anxiety, depressed mood and positive wellbeing among different items. Interestingly, the overall scores of patients were comparable to those of a controlled age-matched population [47]. Of note, almost all of the patients had received ante- and post-natal treatment.

## 4. Conclusions

In the light of literature, it is likely that not treating a maternal infection in pregnancy represents a loss of chance for the fetus, and later the infant. Is it fair to keep pregnant women uninformed about the disease and its prevention and to let fetal infection undiagnosed and untreated?

We recently published with US colleagues a paper depicting the differences in managing congenital toxoplasmosis between the USA and France [50]. In the United States, a pregnant woman will be abruptly told that fetal ultrasound has revealed severe neurological malformations due to toxoplasmic infection, a disease she was, in the majority of cases, totally unaware of. In France, she would have been informed and monthly tested; infection would have been rapidly diagnosed and promptly treated. Eventually antenatal diagnosis would have been performed reassuring her, if negative, or allowing treatment reinforcement. Would antenatal treatment have changed the fetal outcome? Despite the lack of randomized controlled trials, we have now enough evidence to say that antenatal treatment rapidly introduced after maternal infection reduce the risk of fetal infection and the severity of the disease. For care providers, given the accurate tests that are available and the possibility of antenatal diagnosis allowing for treatment adjustment, the absence of screening raises an ethical problem. The lack of consensus with regard of mass screening is a source of confusion placing both doctors and parents to be in a difficult position, especially at a time when the internet has totally changed the patient’s attitude and their relationship with doctors [51]. In the absence of systematic screening, tests could be prescribed during pregnancy, either for medical reason or at women request, and positive results will induce anxiety. In the absence of well-experienced laboratories that are able to estimate the date of infection, under informed doctors will be unable to adequately counsel the parents. Such situation can end-up with an undue pregnancy termination. In the USA, 20% of pregnant women chose pregnancy termination when they are told that they have positive IgM without waiting for confirmation of fetal infection [52]. Contrary to what has been put forward, mass screening with qualified reference laboratories and well informed doctors reduces the number of pregnancy terminations. Many pregnant women who are referred to our center are considering abortion as they were persuaded that their fetus would present with severe malformations. They feel reassured and totally reconsider their decision when told that in our experience based on over 3000 infections during pregnancy, terminations for neurological abnormalities were warranted in less than 1% of cases (personal communication) and that the long term outcome of infected babies is rather good [47]. Today, CT is a neglected disease found in many countries. The main reason is certainly the lack of estimation about the burden of the disease. Indeed, without precise information on the incidence and severity of CT, it is difficult for policy makers to rank this disease among public health priorities. A study qualifying the burden of CT in Belgium identified major data gap in terms of sequelae, intrauterine mortality, termination of pregnancy and late onset of sequelae. A decrease in DALYs was also observed when prevention program was implemented [53].

Lack of consensus puts CT in a “shadow area” where care providers are seeking for guidelines for reassuring pregnant women who sometimes live in a tragedy. The only way to have an accurate estimation on the burden of the disease, without bias or underreporting, and a clear view of its outcome is to implement a mass screening.

Screening to know if it is worth screening.
